# Impact of ageing on the response and repertoire of influenza virus-specific CD4 T cells

**DOI:** 10.1186/1742-4933-11-9

**Published:** 2014-05-13

**Authors:** Kathleen G Lanzer, Lawrence L Johnson, David L Woodland, Marcia A Blackman

**Affiliations:** 1 Trudeau Institute, 154 Algonquin Ave, Saranac Lake, NY 12983, USA; 2 Keystone Symposia, 160 US Highway 6, Suite 200, Silverthorne, CO 80498, USA

**Keywords:** T cell receptor repertoire, Influenza virus, CD4 T cells, Thymectomy, Epitope, Cytokine, Ageing, Mouse model

## Abstract

**Background:**

Ageing has been shown to reduce CD8 T cell repertoire diversity and immune responses against influenza virus infection in mice. In contrast, less is known about the impact of ageing on CD4 T cell repertoire diversity and immune response to influenza virus infection.

**Results:**

The CD4 T cell response was followed after infection of young and aged C57BL/6 mice with influenza virus using a tetramer specific for an immunodominant MHC class II epitope of the influenza virus nucleoprotein. The appearance of virus-specific CD4 T cells in the lung airways of aged mice was delayed compared to young mice, but the overall peak number and cytokine secretion profile of responding CD4 T cells was not greatly perturbed. In addition, the T cell repertoire of responding cells, determined using T cell receptor Vβ analysis, failed to show the profound effect of age we previously described for CD8 T cells. The reduced impact of age on influenza-specific CD4 T cells was consistent with a reduced effect of age on the overall CD4 compared with the CD8 T cell repertoire in specific pathogen free mice. Aged mice that were thymectomized as young adults showed an enhanced loss of the epitope-specific CD4 T cell response after influenza virus infection compared with age-matched sham-thymectomized mice, suggesting that a reduced repertoire can contribute to impaired responsiveness.

**Conclusions:**

The diversity of the CD4 T cell repertoire and response to influenza virus is not as profoundly impaired by ageing in C57BL/6 mice as previously shown for CD8 T cells. However, adult thymectomy enhanced the impact of ageing on the response. Understanding the impact of ageing on CD4 T cell responses to influenza virus infection is an important prerequisite for developing better vaccines for the elderly.

## Background

It is well-established that ageing is associated with a decline in immune function [1,2]. For example, the elderly are highly susceptible to influenza virus infection and respond poorly to influenza vaccines [3]. Although most influenza vaccines elicit protective antibodies, this is of limited long-term protection as the virus changes its coat proteins to avoid neutralizing antibodies. Thus, there is an ongoing effort to develop vaccines that elicit cellular immunity in response to internal, conserved proteins, making them more universal and cross protective. An additional impetus for developing vaccines that elicit cell-mediated immunity is the finding that T cells are a better immune correlate of protection for the elderly against influenza virus than antibody [4].

A major contributing factor to impaired cellular immunity in the elderly is a decline in T cell repertoire diversity. Thymic involution with age results in the export of fewer naïve T cells to the periphery. This reduction in the influx of naïve T cells, coupled with an increase in memory cells due to accumulating antigen experience, enhanced homeostatic proliferation to compensate for reduced naïve T cell production, peripheral selection, and the development of T cell clonal expansions (TCEs) leads to the progressive dominance of numbers of memory compared to naïve T cells in aged individuals [5-9]. A consequence is reduced repertoire diversity among both naïve and memory T cells [10-13]. Decline in CD8 T cell repertoire diversity has been clearly associated with impaired responses to new infections, including influenza virus [11,14-17].

We previously examined the CD8 T cell response to influenza virus infection in aged mice [11]. Consistent with other studies [18-21], the aged mice had delayed viral clearance. In addition, the results showed striking perturbation in the repertoire of responding CD8 T cells in aged mice, with profound shifts in epitope immunodominance and restricted T cell receptor repertoire diversity, in some cases resulting in a “hole” in the repertoire of T cells specific for an immunodominant MHC class I-restricted epitope to influenza nucleoprotein (NP_366–374_/D^b^) [11]. The absence of a primary response to NP translated into a poor response against viral challenge.

Here we have examined the CD4 T cell response to de novo infection with influenza virus in young and aged C57BL/6 mice, using a tetramer specific for an immunodominant CD4 T cell epitope, NP_311–324_/IA^b^. The results show that the response is delayed, but eventually attains numbers and cytokine responses comparable to young mice. Consistent with this, there was little repertoire perturbation of NP-specific CD4 T cells compared to that previously described for NP-specific CD8 T cells. However, the response in thymectomized mice was severely reduced compared to age-matched sham thymectomized mice. These studies are the first to examine the impact of ageing on the CD4 T cell response to a defined influenza virus epitope in a mouse model.

## Results and discussion

### The epitope-specific CD4 T cell response to influenza virus infection is delayed in aged mice

**Figure 1:**
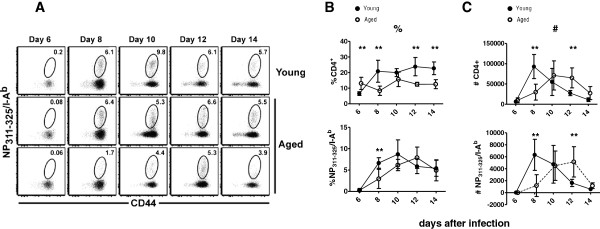
**The appearance of antigen-specific CD4 T cells in the lung airways after primary infection with influenza virus is delayed in aged mice.** Young and aged mice were infected with 3,000 EID_50_ ×-31 influenza virus and the bronchoalveolar lavage (BAL) was assessed at 6, 8, 10, 12 and 14 days post infection for CD4 T cells and Flu NP-specific CD4 T cells. Panel **A** shows representative NP_311–325_/IA^b^ tetramer staining. All data are gated on CD4 T cells. The number in the upper right of each plot indicates the per cent of tetramer^+^ cells among the activated (CD44^high^) CD4 T cells. Panel **B** shows the percent of CD4 T cells and the percent of NP_311–325_/IA^b^-specific cells in the BAL at the indicated timepoints after infection. Panel **C** shows the absolute number of CD4 T cells and NP_311–325_/IA^b^-specific cells in the BAL at the indicated timepoints after infection. The mean ± SD are shown and the data are representative of 2 independent experiments with 5–8 mice per time point. Data were analyzed by unpaired t-test (**p < 0.005).

The impact of ageing on epitope-specific CD4 T cells responding to influenza virus infection in C57BL/6 mice has not previously been determined. To address this issue, we took advantage of a tetramer that recognizes a dominant MHC class II IA^b^ epitope in the influenza virus nucleoprotein NP_311–325_[22-24]. Young (8–10 weeks old) and aged (18–22 months old) mice were infected with influenza virus, and total and NP_311–325_/IA^b^-specific CD4 T cells elicited in the lung airways at 6, 8, 10, 12 and 14 days following infection were enumerated. Representative staining with the tetramer is shown in Figure 1A. Kinetic analysis of the response of CD4 T cells after infection are presented as percent of total and epitope-specific CD4 T cells in the lung airways (Figure 1B) and as absolute numbers of total and epitope-specific CD4 T cells in the lung airways (Figure 1C). The results show the antigen-specific CD4 T cell response is delayed in reaching its peak in aged mice relative to young adults. Although the percent and number of NP_311–325_/IA^b^-specific CD4 T cells in aged mice are significantly lower at day 8 (p < 0.05, t test), they are not statistically different at day 10. Furthermore, the number of cells in aged mice is statistically higher at day 12, as the number of cells in young mice declines (Figure 1C). Thus, rather than an absolute defect in the epitope-specific response in aged mice, the response is kinetically delayed. Such kinetics are consistent with delayed viral clearance, which has been shown to occur in aged mice [19,25,26] and data not shown.

**Figure 2:**
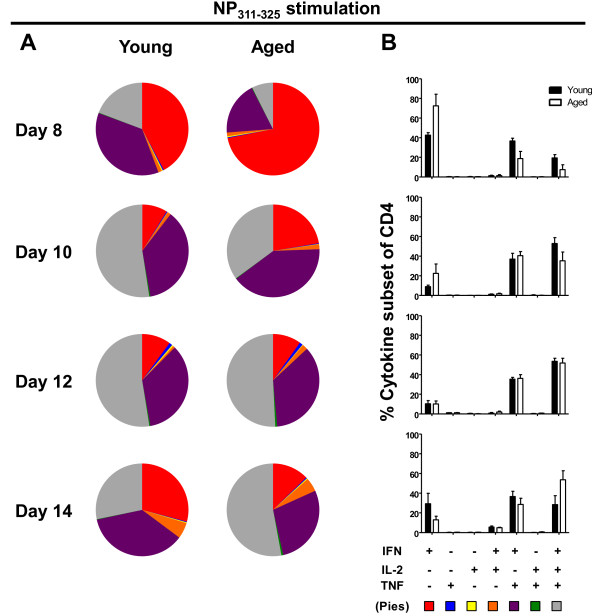
**The cytokine response of influenza NP-specific CD4 T cells is delayed in aged compared with young mice.** Lung airway cells obtained by bronchoalveolar lavage from young and aged mice were analyzed at days 8, 10, 12 and 14 following i.n. infection with 3,000 EID_50_ ×-31 for production of cytokines following *in vitro* stimulation with the NP_311–325_ peptide. Cytokine-producing cells (IFN-γ, TNF-α and/or IL-2) within the CD4^+^CD44^high^ population were divided into seven subpopulations based on their production of these cytokines in combination (refer to color code at the bottom of Panel **B)**. The relative contribution of each of these subpopulations to the responding T cell population was determined as depicted in the pie charts in panel **A**. The bar charts in panel **B** show the frequency of each cytokine subpopulation out of the total responding CD4 T cell population. Data are representative of 2 independent experiments with 5–8 mice per time point.

CD4 T cells play multiple roles in responding to influenza virus infection, including helper function for B cells and CD8 T cells as well as direct effector functions involving IFNγ and/or cytotoxicity (reviewed in [27]. To assess the impact of ageing on the function of NP-specific CD4 T cells, we examined the cytokine secretion profile, including IFNγ, TNFα and IL-2, of lung airway CD4 T cells at 8, 10, 12 and 14 days after infection. CD4 T cells that secrete multiple cytokines are termed polyfunctional and are associated with improved protection after vaccination or secondary viral challenge [28,29]. In Figure 2A, the pie charts show the relative contribution of each cytokine population to the response, in terms of single cytokine producers (IFNγ, TNFα or IL-2 alone), dual cytokine producers (IFNγ + IL-2, IFNγ + TNFα, or TNFα + IL-2), or triple cytokine producers (IFNγ, TNFα and IL-2) (Figure 2A). The bar graphs in Figure 2B show the frequency of each cytokine subset out of the total NP tetramer CD4 T cell population. It can be seen that at days 8 and 10, although aged mice have a higher frequency of IFNγ single positive producers, they have a lower frequency of triple-cytokine producers (IFNγ + IL-2 + TNFα) compared with young mice. By day 12 however, the frequency of triple-cytokine producers in aged mice has reached comparable levels in young mice, and continues to stay high at day 14, at which time the response of the young mice has started to decline. The frequencies of the double IFNγ + TNFα cytokine-producing cells are relatively comparable between young and aged mice at days 10, 12 and 14. Together the data suggest that the CD4 T cells are kinetically delayed in their response.

The observation that the response of CD4 T cells in aged mice is not absolutely defective but is delayed is consistent with findings in elderly humans, in which relatively normal CD4 T cell responses to influenza are observed. However, it has also been found that the responding CD4 T cells were poorly maintained in humans and the development of a memory response was impaired [30,31]. In our studies, CD4 memory T cells established after influenza infection of aged mice maintained function at least for one month (data not shown). More extensive analysis of long-term maintenance of memory is ongoing in our lab.

A major age-associated defect for CD4 T cells has been shown to be reduced IL-2 production [32,33]. However, the NP-specific CD4 T cells examined here in young mice were not strong IL-2 producers (Figure 2). In addition, whereas cytolytic CD4 T cell effectors have been shown to be generated *in vivo* at the site of influenza virus infection [34], the NP-specific cells examined in this study in young mice did not have cytotoxic activity (data not shown). Rather, they were strong polyfunctional cytokine secretors. IFNγ has been shown to play an important role in expansion and trafficking of CD4 and CD8 T cells to the lung [35], and trafficking has been shown to be delayed in aged mice [20], consistent with our data.

### What is the impact of ageing on the T cell repertoire of NP-specific CD4 T cells?

**Figure 3:**
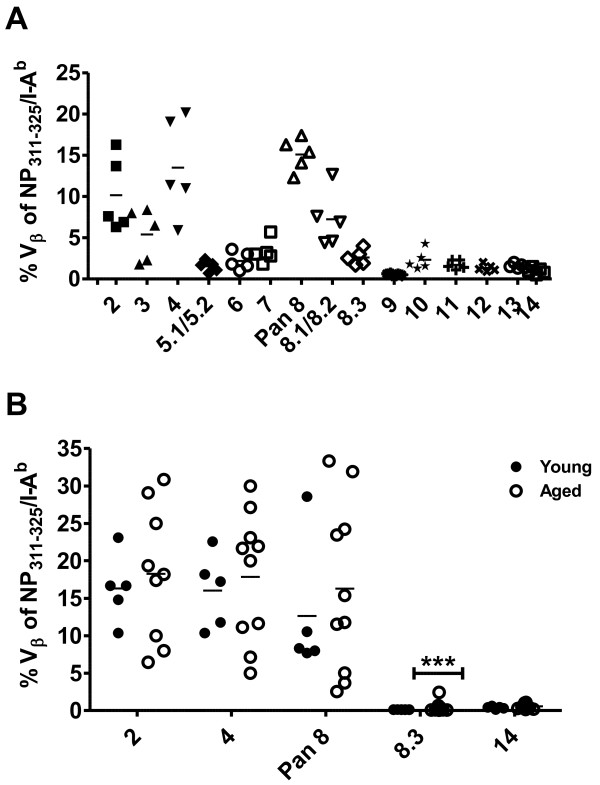
**The T cell receptor Vβ repertoire of influenza NP-specific young and aged CD4 T cells.** Ten days after i.n. infection with 3,000 EID_50_ ×-31 influenza virus, the repertoire of NP_311–325_/IA^b^-specific CD4 T cells in the lungs of mice was assessed by T cell receptor Vβ staining of tetramer-positive CD4 T cells. Panel **A** shows repertoire analysis of NP_311–325_/IA^b^-specific CD4 T cells from the lungs of individual influenza virus-infected young mice (n = 5) using the full panel of anti-Vβ antibodies. Pan 8 signifies Vβ8.1, 8.2 and 8.3. Values for each Vβ are represented by a different symbol. Panel **B** shows selected T cell receptor Vβ repertoire analysis of NP_311–325_/IA^b^-specific CD4 T cells from the lungs of individual young (closed circles, n = 5) and aged (open circles, n = 10) mice 12 days post infection. Variances were compared by F test (***p < 0.0005). Group means are indicated by bars.

We next addressed whether the delayed appearance of epitope-specific CD4 T cells after influenza virus infection of aged mice was associated with perturbations in the T cell receptor repertoire, as we have described for CD8 T cells [11]. We first characterized the NP-specific CD4 T cell receptor Vβ repertoire in detail among individual young mice using the entire panel of T cell receptor Vβ antibodies (Figure 3A). We then selected 5 of the antibodies to use for characterization of the response of individual young and aged mice, focusing on Vβ2, Vβ4 and pan Vβ8 (Vβ8.1, 8.2 and 8.3) as highly represented Vβs, and Vβ8.3 and Vβ14 as under-represented Vβs in the repertoire of young mice. The analysis showed that the Vβ usage of NP-specific CD4 T cells was more variable among individual aged compared with young mice, but except for Vβ8.3 the difference was not statistically significant (Figure 3B). Taken together, the data show little impact of age on the NP-specific CD4 T cell repertoire, in contrast to that previously observed for the NP-specific CD8 T cell repertoire [11,20,21]. This difference prompted us to examine the impact of ageing on total (non-antigen-specific) peripheral CD4 and CD8 T cell pools.

### Is there a differential impact of ageing on the CD4 and CD8 T cell pools?

**Figure 4:**
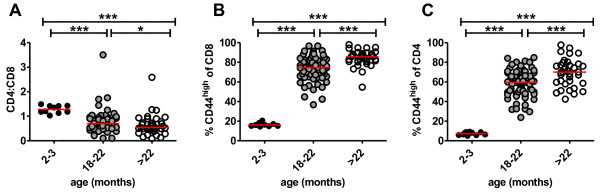
**Age-associated changes in distribution of peripheral CD4 and CD8 T cells.** Peripheral blood T cells from individual young (2–3 months of age, n = 10) and aged (18–22 months of age, n = 66 and >22 months of age, n = 36) mice were analyzed for distribution of CD4, CD8 and CD44. The median values of each population are shown with red bars. Panel **A** shows the CD4:CD8 ratio in young and aged mice. Data were analyzed by Mann–Whitney test (*p < 0.05, ***p < 0.0005). Panel **B** shows the age-related differences in the proportion of CD44^high^ cells among CD8 T cells. Panel **C** shows the age-related differences in the proportion of CD44^high^ cells among CD4 T cells. Data in panels **B** and **C** were analyzed by Mann–Whitney test (***p < 0.0005).

The absence of dramatic age-associated T cell receptor repertoire perturbations in the CD4 T cell response of aged mice to NP_311–324_/IA^b^, in contrast to previous studies showing a strong impact of aging on the T cell receptor repertoire of influenza-specific CD8 T cells [11], prompted us to examine the effect of age on the general distribution of CD4 and CD8 T cell pools by analyzing peripheral blood T cells in specific pathogen free mice. It has been shown in some, but not all, studies that the ratio of CD4:CD8 T cells declines progressively with age [8,36-40]. Our data show a distinct shift in the CD4:CD8 ratio from greater than 1 in young mice to less than 1 in aged (18 to >22 months of age) mice (Figure 4A).

We also examined the proportions of naïve and memory cells in the peripheral blood among CD4 and CD8 T cells to determine if there was a general difference of the effect of ageing on CD4 and CD8 T cells. CD44 is a cell surface marker used to distinguish naïve (CD44^low^) from activated/memory (CD44^high^) T cells [41]. The data show that the proportion of CD44^high^ cells dramatically increased comparably with age among both CD4 and CD8 T cells (Figure 4B, C), confirming no preferential generalized effect of age on CD8 T cells.

**Figure 5:**
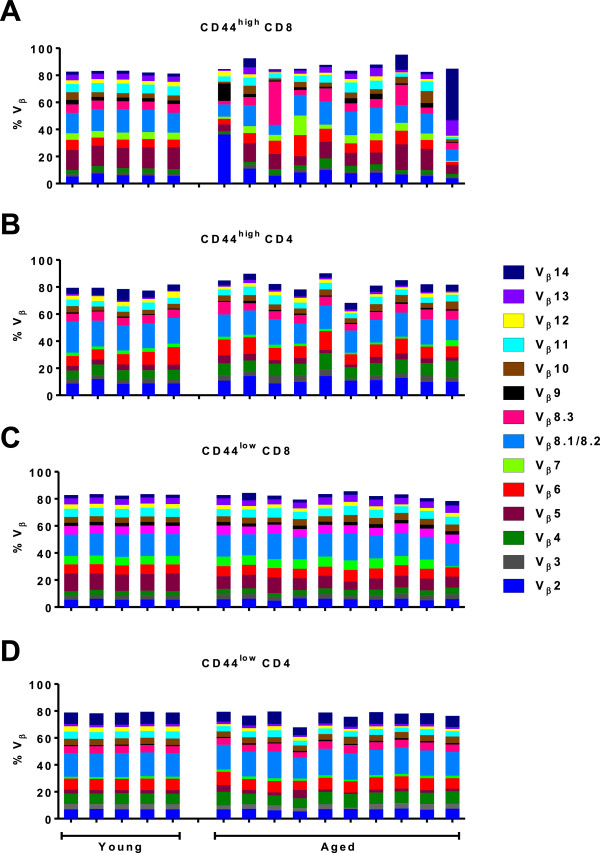
**Age-associated changes in T cell receptor Vβ distribution in CD4 and CD8 naïve and activated/memory T cells in specific pathogen free mice.** T cell receptor Vβ usage was assessed among distinct populations of CD44^high^ (activated/memory) or CD44^low^ (naïve) CD4 and CD8 T cells in individual young and aged mice. The proportion of cells from 5 young or 10 aged mice bearing individual T cell receptor Vβ elements was determined by staining with a panel of anti-Vβ antibodies, and is represented by different colors, which are the same order in the key and individual samples. Panel **A** shows the Vβ distribution among CD44^high^ CD8 T cells. Panel **B** shows the Vβ distribution among CD44^high^ CD4 T cells. Panel **C** shows the Vβ distribution among CD44^low^ CD8 T cells. Panel **D** shows the Vβ distribution among CD44^low^ CD4 T cells. Statistical analysis is described in the text.

Finally, to assess overall perturbations in the aged T cell repertoire among CD4 and CD8 T cells, we analyzed T cell receptor Vβ usage among activated/memory (CD44^high^) and naïve (CD44^low^) CD4 and CD8 T cells in young and aged specific pathogen free mice (Figure 5), using a panel of fourteen well-characterized T cell receptor Vβ antibodies. The percentages of each Vβ from individual young (left set of 5 bars) and aged (right set of 10 bars) mice are represented by different colors presented in the same order as the color key in the right of the Figure. Using the data in Figure 5 we asked whether there were significant differences with regard to the usage of different Vβs in various comparisons: i.e., young vs aged, CD4 vs. CD8 T cells, and activated/memory (CD44^high^) vs naïve (CD44^low^) T cells. To make those comparisons we calculated statistics (mean and variance) for each Vβ represented by a different colored bar in the rainbow plots within each of the eight groups (young or aged) shown in the four panels A-D in Figure 5. For every Vβ, we quantified its variability in usage within each of the eight groups by its *variance*. This allowed us to determine whether a given Vβ exhibited significantly more variable usage (measured by its variance) within one group than within another. Thus, we conducted a group of F tests to determine whether corresponding pairs of variances for the same Vβ differed significantly.

Those F tests revealed that among CD44^high^ CD8 T cells, almost all (13 of 14) variances in Vβ usage in aged T cells differed significantly from the variance for the corresponding Vβ in young mice (Panel A), whereas among CD44^high^ CD4 T cells (Panel B), only 2 (Vβ5 and Vβ7) of 14 such variance comparisons were statistically significant by F test. Thus, using Fisher’s exact test on the number of significant differences (2 of 14 vs. 13 of 14) we conclude that aged CD44^high^ CD8 T cells exhibit many more instances of perturbation of their Vβ usage, i.e., significant differences in variance when gauged against Vβ usage in CD44^high^ cells from young mice as a reference, than do aged CD44^high^ CD4 T cells gauged against Vβ usage in CD44^high^ cells from young mice (p < 0.0001). That difference is evident visually by inspection of the rightmost rainbow plots (aged mice) in panel A (CD8 T cells) and panel B (CD4 T cells) compared with the corresponding leftmost rainbow plots (young mice) in each case.

To assess variability in Vβ usage directly among CD44^high^ CD8 and CD4 T cells from aged mice only, we compared variances for each of the 14 Vβs in the two T cell subsets (rightmost plots in panels A and B) by F tests. When compared directly with each other, the variance for every Vβ except Vβ3 and Vβ5 in CD44^high^ CD8 T cells differed significantly from the same Vβ in CD44^high^ CD4 T cells. Thus, in all but two of 14 Vβ, usage was more perturbed in CD44^high^ CD8 T cells than in CD44^high^ CD4 T cells.

An analogous analysis was performed on naïve (CD44^low^) CD8 and CD4 T cells from aged mice (panels C and D). Here, in contrast to the analysis with CD44^high^ T cells, there were only three instances of significant differences in variance between CD8 and CD4 T cells from aged mice, Vβ3, Vβ5, and Vβ7.

Together, these analyses show that there is significantly more variability in the usage of Vβs in CD44^high^ CD8 T cells from aged mice than in the CD44^high^ CD4 T cell population in aged mice. Thus, despite age-associated perturbations in both CD4 and CD8 T cells in terms of an increase in the proportion of activated/memory CD44^high^ cells (Figure 4), there is less age-associated perturbation in Vβ usage in aged CD44^high^ CD4 compared to CD8 T cells. This is in agreement with most reports regarding the impact of ageing on the overall CD4 T cell repertoire. For example, several studies in both mouse and human show that the CD4 T cell repertoire is less profoundly perturbed with age compared to the CD8 repertoire [42-47]. In contrast, other studies reported repertoire perturbations and the development of TCE in the human CD4 T cell repertoire as well as the CD8 T cell repertoire, although TCEs were smaller within the CD4 T cell subset [48,49].

The conflicting results in separate studies may be a consequence of the age of individuals analyzed. It has been shown that repertoire diversity in CD4 T cells is well-maintained between the ages of 25 and 60 years. However, after age 70, there is a sharp decline in CD4 repertoire diversity, with a greater than 100-fold constriction [12,50]. Thus, the studies which included individuals over 70 years of age showed perturbations in the CD4 repertoire. As the 18–22 month old mice studied here are roughly equivalent to 56–69 years of human age [51], it is possible that CD4 repertoire perturbations would be more profound in older mice.

### Thymectomy enhances age-associated perturbation of peripheral T cells and deficiency in the CD4 T cell response to NP

**Figure 6:**
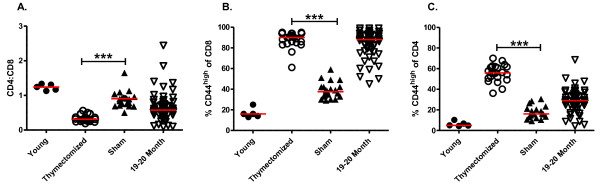
**Thymectomy enhances age-associated changes in the CD4:CD8 ratio and distribution of naïve and memory T cells.** The peripheral blood from young mice, thymectomized and sham thymectomized 15 month old mice, and aged (19–20 month old) euthymic mice was analyzed with antibodies for CD4, CD8 and CD44. The median values of each population are shown with red bars. Panel **A** shows the CD4:CD8 ratio. Data were analyzed by Mann–Whitney Test (***p < 0.0005). Panel **B** shows the percent CD44^high^ among CD8 T cells. Panel **C** shows the percent of CD44^high^ among CD4 T cells. Data in panels **B** and **C** were analyzed by Mann–Whitney test (***p < 0.0005).

The lesser impact of age on the repertoire of CD4 compared with CD8 T cells prompted us to examine CD4 T cells after influenza infection of thymectomized mice. Thymus involution with age results in reduced seeding of naïve T cells to the periphery and thus makes a major contribution to age-associated loss of repertoire diversity in the peripheral T cell repertoire. This aspect of ageing can be accelerated with adult thymectomy [52], which has been used as an experimental model in mice [53]. Thus, adult mice were thymectomized or sham-thymectomized at four weeks and allowed to age for 9–14 months, at which time peripheral blood was analyzed to determine the impact of thymectomy on the CD4:CD8 ratio and the ratio of CD44^high^ and CD44^low^ cells within the CD8 and CD4 peripheral T cell pools (Figure 6). The data show that the thymectomized mice had a dramatically lower CD4:CD8 ratio (Figure 6A) compared with young mice and age-matched sham-thymectomized mice, and that the ratio was decreased and more homogeneous compared to the aged euthymic mice. Thymectomy also enhanced the transition to a CD44^high^ memory phenotype of both CD8 (Figure 6B) and CD4 (Figure 6C) T cells, compared to young mice and age-matched sham-thymectomized mice. Thus, thymectomy enhanced the impact of ageing on both peripheral CD4 and CD8 T cell pools. Particularly important for this study, the thymectomized mice had a greater conversion of CD4 T cells to a memory phenotype than the 19–20 month old euthymic mice (Figure 6C).

**Figure 7:**
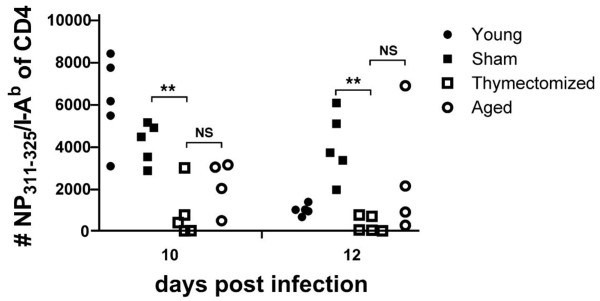
**Thymectomy reduces the response of NP-specific CD4 T cells in the lung airways of influenza virus-infected mice.** Young mice, aged mice (19–20 months old) and thymectomized and sham thymectomized 10 month old mice (9 months after thymectomy) were infected with 3,000 EID_50_ ×-31 influenza virus and the bronchoalveolar lavage (BAL) was assessed at 10 and 12 days post infection for total CD4 T cells and Flu NP-specific CD4 T cells. The absolute number of NP_311–325_/IA^b^-specific CD4 T cells in the BAL of individual mice from a representative experiment of two is shown. Data were analyzed by unpaired t-test with Welch correction (**, p < 0.005; NS, not significant).

To determine whether the accelerated perturbation of the peripheral CD4 T cell pool in thymectomized mice translated into greater disruption of the CD4 T cell response to influenza virus infection, we followed the response kinetically in thymectomized, sham-thymectomized, young and aged mice, using the NP_311–325_/IA^b^ class II tetramer. The response in terms of numbers of epitope-specific CD4 T cells was generally reduced in thymectomized mice compared to aged mice, but due to variability among individual aged mice, the differences were not statistically significant (Figure 7). A more relevant comparison is between thymectomized and sham-thymectomized mice of the same age. As seen in Figure 7, the response of individual mice at 10 and 12 days post infection show that the number of NP-specific CD4 T cells elicited in the bronchoalveolar lavage was greatly impaired in thymectomized mice relative to age-matched sham-thymectomized mice. This re-enforces the conclusion that thymectomy enhances the effects of ageing on CD4 T cell responses. Whether this is a direct consequence of reduced production of naïve T cells is unclear, as reduced thymic export has also been associated with accelerated decay of naïve CD4 T cells in the periphery and/or peripheral selection [54,55]. The decline in CD4 T cell response to influenza infection in thymectomized mice is also consistent with the observation that mice retain residual thymic function throughout their lifespan [56]. We were unable to directly examine the T cell Vβ usage of the NP-specific cells, due to the paucity of responding cells.

The limited effect of age on the CD4 T cell repertoire to influenza contrasts with previous data showing a profound impact of ageing on the influenza-specific CD8 T cell repertoire, in some cases leading to the development of “holes” in the repertoire. This difference could be a consequence of differences in thymic output or differences in homeostatic maintenance of CD4 and CD8 T cells with age. For example, it has been shown that T cell receptor excision circles (TRECs), which are a measure of thymic output, are retained longer in the CD4 compared with the CD8 pool of T cells [57]. Alternatively, the difference in age effects on the CD4 and CD8 T cell influenza-specific repertoire may be epitope dependent, rather than a general difference between CD4 and CD8 T cells, as we were only able to examine CD4 T cells specific for a single epitope.

## Conclusions

In the current studies, we have analyzed the impact of ageing on the response and T cell repertoire of CD4 T cells specific for an immunodominant influenza virus nucleoprotein epitope, NP_311–325_, in C57BL/6 mice. First, the data show that the response in the lung airways following influenza virus infection is delayed but eventually attains comparable numbers and cytokine secretion patterns as young mice. In striking contrast, the response of NP-specific CD4 T cells in mice that were thymectomized at four weeks of age and analyzed 9–14 months later was virtually ablated. This result supports the concept that there is residual thymic export of naïve CD4 T cells in aged mice [58]. Second, consistent with the findings of others, the impact of ageing on the overall T cell repertoire of CD4 T cells was less profound than that on CD8 T cells [42-47]. Specifically, the current data show that the repertoire of influenza NP-specific CD4 T cells, assessed by Vβ usage, was only mildly perturbed in aged mice. Thus, there was less of an effect of ageing on the influenza-specific CD4 T cell repertoire than we had previously shown for the CD8 T cell repertoire [11]. Whether this was due to an overall greater effect of ageing on the CD8 T cell repertoire, or merely an epitope-specific difference as a consequence of the few influenza virus-specific epitopes analyzed, cannot be determined from these data.

In summary, these data are, to our knowledge, the first to examine the impact of ageing on the CD4 T cell response to a defined influenza virus epitope in mice using an MHC class II tetramer. Understanding the impact of ageing on CD4 T cells is important for vaccine design because it has recently been shown that pre-existing influenza-specific CD4 T cells correlate with disease protection against influenza challenge in humans [59].

## Materials and methods

### Mice, viruses, and infections

C57BL/6 mice were obtained from the Trudeau Institute animal facility and maintained under specific pathogen-free conditions. Often, but not always, impaired immune function has been attributed to the presence of large TCEs in the CD8 population [11,45]. To avoid this complicating factor, peripheral blood lymphocytes of all aged mice were prescreened for major CD8 T cell Vβ expansions, and those that exhibited TCR Vβ8, Vβ7 or Vβ8.3 staining ± 4 SD over that observed with young C57BL/6 mice were omitted from the study. Mice were thymectomized or sham-thymectomized at 4 weeks of age and maintained until 10–15 months of age. Influenza virus A/HK-×31 (×31, H3N2) was grown, stored, and titered as previously described [60]. Young (8–10 weeks old) and aged (18–22 months old) mice were anesthetized with 2,2,2-tribromoethanol (200 mg/kg) and infected with 3,000 EID_50_ ×-31. All animal procedures were approved by the Institutional Animal Care and Use Committee at Trudeau Institute.

### Tissue harvest and flow cytometry

Blood samples were obtained by mandibular bleed, and the bronchoalveolar lavage was obtained and cells were processed as described previously [61]. Single cell suspensions were incubated with Fc-block (anti-CD16/32) for 15 minutes on ice followed by staining with MHC class I and II peptide tetramers specific for influenza virus (NP_366–374_/D^b^ and NP_311–324_/IA^b^) for 1 hour at room temperature. Tetramers were generated by the Trudeau Institute Molecular Biology Core (NP_366–374_/D^b^ and NP_311–324_/IA^b^) or obtained from the NIH Tetramer Core Facility (NP_311–324_/IA^b^). Tetramer-labeled cells were incubated with antibodies to surface proteins (CD4, CD8, CD44, or T cell receptor anti-Vβ antibodies that included Vβ2, Vβ3, Vβ4, Vβ5.1/5.2, Vβ6, Vβ7, Vβ8, Vβ8.1/8.2, Vβ8.3, Vβ9, Vβ10, Vβ11, Vβ12, Vβ13 and Vβ14) for 30 minutes on ice. Antibodies were purchased from BD Biosciences, eBioscience and BioLegend. Samples were run on a FACS Canto II flow cytometer (BD Biosciences) and data were analyzed with Flow Jo software (TreeStar).

### Intracellular cytokine staining

For measurement of cytokine production, single cell suspensions were incubated with NP_311–324_/IA^b^ or control peptides as previously described [62]. Cells were stained for surface markers, fixed and permeabilized (CytoFix/CytoPerm kit, BD Biosciences), and stained for intracellular cytokines with antibodies to IFN-γ, TNFα and IL-2 for 30 minutes on ice. Samples were run on a FACS Canto II flow cytometer (BD Biosciences) and data were analyzed with Flow Jo software (TreeStar).

### Statistical analysis

Methods for statistical analysis, performed with Prism 5 (Graphpad software) or Microsoft Excel (F tests), are detailed in the text or Figure legends.

## Abbreviations

TCE: T cell clonal expansions; TCR: T cell receptor; Vβ: The variable region of the T cell receptor beta chain; NP: Nucleoprotein.

## Competing interests

The authors’ declare that they have no competing interests.

## Authors’ contributions

KL, DW and MB designed the study; KL generated and analyzed the data; KL, LJ, DW and MB interpreted the data and wrote the manuscript; LJ and KL did the statistical analysis. All authors read and approved the final manuscript.
